# Novel lead structures with both *Plasmodium falciparum* gametocytocidal and asexual blood stage activity identified from high throughput compound screening

**DOI:** 10.1186/s12936-017-1805-0

**Published:** 2017-04-13

**Authors:** Wei Sun, Xiuli Huang, Hao Li, Gregory Tawa, Ethan Fisher, Takeshi Q. Tanaka, Paul Shinn, Wenwei Huang, Kim C. Williamson, Wei Zheng

**Affiliations:** 1 National Center for Advancing Translational Sciences, National Institutes of Health, 9800 Medical Center Drive, Bethesda, MD 20892 USA; 2 Laboratory of Malaria and Vector Research, National Institute of Allergy and Infectious Diseases, Bethesda, MD USA; 3 Department of Microbiology and Immunology, Uniformed Services University of the Health Sciences, Bethesda, MD 20814 USA

**Keywords:** Malaria, Transmission blocking, Gametocyte, Asexual parasite, Blood stage, Dual-efficacy, High throughput screen, Cheminformatics, Novel structures, Dark chemical matter

## Abstract

**Background:**

Blocking malaria transmission is an important step in eradicating malaria. In the field, transmission requires the production of sexual stage *Plasmodium* parasites, called gametocytes, which are not effectively killed by the commonly used anti-malarials allowing individuals to remain infectious after clearance of asexual parasites.

**Methods:**

To identify new gametocytocidal compounds, a library of 45,056 compounds with diverse structures was screened using a high throughput gametocyte viability assay. The characteristics of active hits were further evaluated against asexual stage parasites in a growth inhibition assay. Their cytotoxicity were tested against mammalian cells in a cytotoxicity assay. The chemical scaffold similarity of active hits were studied using scaffold cluster analysis.

**Results:**

A set of 23 compounds were identified and further confirmed for their activity against gametocytes. All the 23 confirmed compounds possess dual-activities against both gametocytes responsible for human to mosquito transmission and asexual parasites that cause the clinical symptoms. Three of these compounds were fourfold more active against gametocytes than asexual parasites. Further cheminformatic analysis revealed three sets of novel scaffolds, including highly selective 4-1H-pyrazol-5-yl piperidine analogs.

**Conclusions:**

This study revealed important new structural scaffolds that can be used as starting points for dual activity anti-malarial drug development.

**Electronic supplementary material:**

The online version of this article (doi:10.1186/s12936-017-1805-0) contains supplementary material, which is available to authorized users.

## Background

Malaria remains a major health problem in underdeveloped countries. In the past decade, mortality rates have dropped significantly owing to the combined efforts of insecticidal bed nets and artemisinin-based combination therapy (ACT) [1, 2], but 214 million estimated malaria cases still occurred worldwide in 2015. The success of ACT has renewed hopes and provided a unique opportunity for researchers to consider new approaches to eliminate malaria [3, 4]. Due to the complex life cycle of parasites, currently no single strategy effectively treats human disease and controls parasite transmission. Malaria vaccine development also continues to be a challenge. RTS, S, the only malaria vaccine approved for use outside of trials, has low efficacy (26–50%) in young children and is being evaluated by the World Health Organization (WHO) [5], who recently announced a pilot roll-out of the vaccine in three African countries.

One of the major hurdles to malaria elimination is the lack of effective agents to prevent and control malaria transmission from mosquito to human. The malaria life cycle requires a mosquito vector ingest sexual stage parasites, called gametocytes, during a human blood meal. In the human host, *Plasmodium falciparum* gametocytes develop through five stages (I–V) over 10–12 days after RBC invasion by a merozoite committed to sexual differentiation. The mature stage V gametocytes then circulate in the peripheral blood for 4–6 days. Once taken up in a blood meal by a mosquito, male and female stage V gametocytes are stimulated to undergo fertilization and form oocysts on the basal surface of the midgut. The infectious form of the parasite, sporozoites, are formed in the oocysts and after maturation they are released and migrate to the salary glands. During a subsequent blood meal the sporozoites are transmitted to humans with the saliva. Most of the current drug development efforts have been devoted to controlling the asexual parasites that are responsible for the disease symptoms in patients. Currently, the only drugs that are active against gametocytes and can block malaria transmission are 8-aminoquinolines such as primaquine. However, 8-aminoquinolines can cause haemolysis in patients with glucose-6-phosphate dehydrogenase (G6PD) deficiency, a highly prevalent genetic condition in malaria-endemic regions [6]. To date, only a few drug candidates in preclinical or clinical stages have the potential to block malaria transmissions [1, 7]. This deficit is partly due to difficulty in producing *P. falciparum* gametocytes in culture, a process that takes at least 12–14 days with very limited yield [8]. This hurdle limits the capacity of malaria gametocytes for compound screening even with the recent development of several high throughput assays [9–13]. Consequently, only limited compound collections have been screened, including two screens of FDA approved drugs collections [14, 15], several screens of MMV Malaria Box library [16], and additionally, three relatively large scale screens of ~10,000 molecules [9, 16, 17]. These initial screens are a good start, but additional novel lead compounds with dual-activities against both gametocytes and asexual parasites are highly needed.

The emergence of anti-malarial resistance is of substantial concern to the malaria community [18]. Recently, artemisinin resistance in *P. falciparum* has spread in Greater Mekong subregion [19]. Current and future drug screens using new chemical entities with novel modes of action, instead of analogs of the previous anti-malarials, will have a better opportunity to address the drug-resistance [20, 21]. In the previous studies, a 1536-well high throughput gametocyte viability assay was developed [11, 22] and used for screening of several known compound libraries, including 1280 compounds in the LOPAC library [11], 4265 approved drugs and 400 from the MMV Malaria Box library [14]. Here, this study reports the results of high throughput screening of 45,056 compounds with diverse structures with the gametocyte viability assay. The results revealed 3 groups of novel structures that actively suppress both gametocytes and asexual parasites.

## Methods

### Cell culture

Asexual parasites of *P. falciparum* strain NF54 were cultured as described previously [23]. Briefly, parasites were maintained in RPMI 1640 medium containing 10% positive human serum + erythrocytes (5% haematocrit), 2.5% sodium bicarbonate and 11 µg/mL gentamicin at 37 °C with 5% CO_2_, 5% O_2_ and 90% N_2_. Gametocyte cultures were set up at 0.1% parasitaemia and on days 9–10 treated with 50 mM *N*-acetylglucosamine (NAG) to block further asexual growth. Stage III–V gametocytes were isolated by Percoll density gradient centrifugation on day 12 and returned to culture for 24 h before being used in the assay [22]. At the time of the assay over 73% of the gametocytes were stage IV or V (Additional file 1). HepG2 cells (ATCC, cat. no. 77400) were cultured in 175-cm^2^ tissue culture flasks with 30 ml growth medium at 37 °C in a 5% CO_2_ and 5% O_2_ humidified atmosphere. Growth medium was made with Dulbecco’s Modified Eagle Medium with 10% fetal bovine serum (FBS). Growth medium was replaced every other day and cells were passaged at 75% confluence.

### Compound library and gametocyte assay screen

Compounds from the Sytravon library (a retired Pharma screening collection that contains a diversity of novel small molecules, with an emphasis on medicinal chemistry-tractable scaffolds) were obtained as powder samples and dissolved in DMSO as 400 and 80 µM stock solutions. Compound screening experiments were performed as previously described [11, 14]. In the primary screen, two concentrations for each compound were tested. No technical and biological replicates were involved. In the follow-up confirmation studies, three biological replicates were tested for each compound in both parasite assays. The positive control was 46 µM of Torin 2 and negative control was DMSO. Briefly, 2.5 μL/well incomplete medium was dispensed into each well of 1536-well plates using the Multidrop Combi followed by 23 nL compound transferring using the NX-TR Pintool (WAKO Scientific Solutions, San Diego, CA). Then, 2.5 μL/well of gametocytes was dispensed with a seeding density of 20,000 cells/well using the Multidrop Combi. The assay plates were incubated for 72 h at 37 °C with 5% CO_2_. After addition of 5 μL/well of 2X AlamarBlue dye (Life Technologies, cat. no. DAL1100), the plates were incubated for 24 h at 37 °C with 5% CO_2_ and then were read in a fluorescence detection mode (Ex = 525 nm, Em = 598 nm) on a ViewLux plate reader (PerkinElmer).

### Asexual stage parasites drug activity assay

Drug activity on asexual stage parasites was tested using a SYBR Green assay as described previously [24]. Briefly, 2.5 μL/well complete culture medium was dispensed into each well of 1536-well plates using the Multidrop Combi followed by 23 nL compound transferring using the NX-TR Pintool. Parasites were diluted to 0.5% parasitaemia in complete culture medium with 2% haematocrit and drugs diluted in DMSO (≤0.5%). The pre-diluted parasites were dispensed into a 1536-well plate (2.5 μL/well). After 72 h incubation under the standard culture conditions, 5 μL/well of lysis buffer containing SYBR Green I was added to the parasite culture and incubated for 30 min at room temperature. The fluorescence of each well was measured in a fluorescence detection mode (Ex = 490 nm, Em = 520 nm) using a ViewLux plate reader (PerkinElmer).

### Human cell line cytotoxicity assay

Drug activity on HepG2 was tested using an AlamarBlue assay as previously described [25]. Briefly, 5 μL/well of HepG2 cells were dispensed with a seeding density of 1000 cells/well using the Multidrop Combi. 23 nL compound was transferred using the NX-TR Pintool. After 72 h incubation under the standard culture condition, 5 μL/well of 2X AlamarBlue dye was added to the cells, the plates were incubated for 2 h at 37 °C with 5% CO_2_ and then were read in a fluorescence detection mode (Ex = 525 nm, Em = 598 nm) on a ViewLux plate reader.

### Data analysis

The primary screen data was analysed using customized software developed internally [26]. IC_50_ values were calculated using the Prism software (Graphpad Software, Inc. San Diego, CA). The Z’ factors of the five HTS screens were 0.46, 0.38, 0.53, 0.55, and 0.62. The Z’ factors of asexual stage parasites and cytotoxicity assays were >0.5. The scaffold cluster analysis was performed based on the chemical scaffold similarity of 32 hits. Hit molecules with similar core structures were grouped into a cluster. Known anti-malarial scaffolds were confirmed in both the asexual and the gametocyte assays.

## Results

### High throughput screening with a gametocyte viability assay identified 32 primary hits

**Fig. 1:**
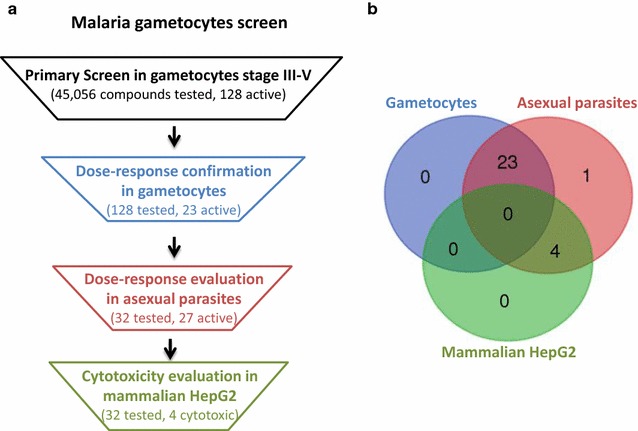
Flowchart of malaria gametocyte viability screens and compound confirmation in gametocyte, asexual parasites and mammalian cell assays. **a** The primary screens of the Sytravon libraries were carried out in malaria *P. falciparum* gametocyte viability assays. A group of 128 hits from the malaria gametocyte screen were selected for confirmation in the same assays. Further, 32 hits were picked for inhibition of asexual parasites growth and tested for cytotoxicity in the mammalian HepG2 cell line. **b** Venn-diagram of confirmed hits with activities against *P. falciparum* gametocytes, *P. falciparum* asexual parasites, and the mammalian HepG2 cells. NCGC00104528 was clustered as active against both gametocytes and asexual parasites in Venn diagram (IC_50_ of 8.91 µM in gametocyte viability assay and IC_50_ of 13.9 µM in asexual parasite growth assays)

**Fig. 2:**
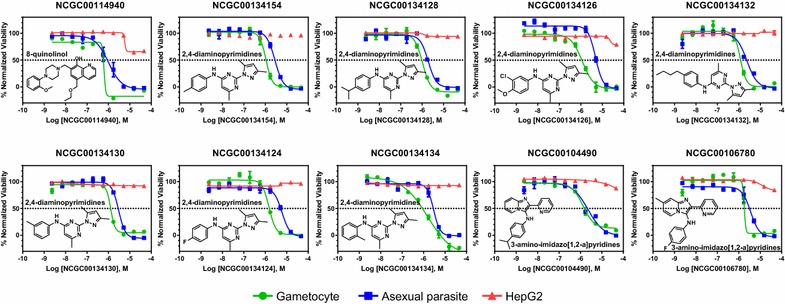
Structures and activities of 10 selected anti-gametocyte compounds. Structures and concentration-response curves of selected lead compounds (NCGC00114940, NCGC00134134, NCGC00134128, NCGC00134154, NCGC00134126, NCGC00134132, NCGC00104490, NCGC00134130, NCGC00134124, and NCGC00106780) determined in the gametocyte viability assay (*green*), asexual parasite growth assay (*blue*) and HepG2 cytotoxicity assay (*red*). Cluster names are included. Data are presented as mean ± SD with n = 3 independent experiments

A total of 45,056 compounds were screened against enriched stage III-V gametocytes using the previously developed high throughput viability assay [11, 14, 22] (Fig. 1a, b). Due to the intrinsic difficulty in producing a large amount of gametocytes, the primary screen was split into five campaigns. To identify compounds with activities in the low micromolar range, each compound at 400 µM (final concentration was 1.84 µM) and 80 µM (final concentration was 0.368 µM) were added to the gametocytes in singlet in the primary screen. These concentrations were selected because the available stock concentrations in the compound library. A set of 128 primary hits that had >50% gametocytocidal activity at either compound concentration were selected for validation in the same gametocyte assays using an 11 point concentration titration ranging from 0.001 to 46 µM. The activity of 32 of these compounds was confirmed and 23 of the 32 had an IC_50_ less than 10 µM with >75% maximum activity (Table 1; Fig. 2).

**Table 1:** Cluster and activity of 32 compounds against *Plasmodium falciparum* NF54 gametocytes, asexual parasites and cytotoxicity of these compounds against HepG2

Cluster	Sample ID	Structure	Gametocyte, IC_50_ (µM)	Asexual, IC_50_ (µM)	HepG2, IC_50_ (µM)	Asexual/Gametocyte
Cluster 1 2,4-diaminopyrimidines	NCGC00134128		1.18 ± 0.17	1.93 ± 0.13	–	1.64
	NCGC00134154		1.19 ± 0.34	2.83 ± 0.31	–	2.38
	NCGC00134126		1.19 ± 0.05	4.78 ± 0.43	–	4.02
	NCGC00134130		1.42 ± 0.24	3.26 ± 0.50	–	2.30
	NCGC00134124		1.55 ± 0.11	5.96 ± 0.70	–	3.85
	NCGC00134158		2.08 ± 0.18	4.70 ± 0.52	–	2.26
	NCGC00134156		2.10 ± 0.73	3.53 ± 0.27	–	1.68
	NCGC00134132		1.29 ± 0.32	2.41 ± 0.32	–	1.87
	NCGC00134136		2.57 ± 0.48	4.12 ± 0.10	–	1.60
	NCGC00134134		1.10 ± 0.16	3.12 ± 0.87	–	2.84
Cluster 2 3-amino-imidazo[1,2-a]pyridines	NCGC00104490		1.29 ± 0.29	2.22 ± 0.35	–	1.72
	NCGC00106055		3.98 ± 1.12	4.18 ± 0.68	–	1.05
	NCGC00106087		3.34 ± 0.51	7.49 ± 4.11	–	2.24
	NCGC00106780		1.82 ± 0.11	3.53 ± 1.21	–	1.94
	NCGC00106091		2.45 ± 0.42	4.51 ± 1.29	–	1.84
	NCGC00106119		2.82 ± 0.30	4.22 ± 0.29	–	1.50
	NCGC00104528		8.91 ± 0.20	13.9 ± 0.93	–	1.56
Cluster 3 4H-chromen-4-ones	NCGC00100599		4.34 ± 0.60	0.34 ± 0.03	–	0.08
	NCGC00101506		4.82 ± 0.21	0.54 ± 0.05	–	0.11
	NCGC00141020		4.86 ± 0.03	1.07 ± 0.37	–	0.22
	NCGC00100597		6.04 ± 1.90	0.63 ± 0.05	–	0.10
Cluster 4 4-1H-pyrazol-5-yl piperidines	NCGC00127017		11.9 ± 2.05	3.44 ± 0.24	25.3 ± 5.41	0.29
	NCGC00126892		31.3 ± 12.8	7.49 ± 0.86	34.4 ± 5.36	0.24
	NCGC00127015		20.3 ± 0.31	3.05 ± 0.27	38.4 ± 0.48	0.15
	NCGC00126987		30.6 ± 1.95	1.47 ± 0.04	38.6 ± 0.42	0.05
	NCGC00124960		~60	35.6 ± 1.77	–	0.59
Cluster 5 8-quinolinol	NCGC00114940^a^		0.62 ± 0.04	1.16 ± 0.57	–	1.87
Cluster 6 3H-imidazo[4,5-b]pyridine	NCGC00134795		4.25 ± 0.64	0.96 ± 0.30	–	0.23
Cluster 7 thieno[2,3-e][1,2,3]triazolo[1,5-a]pyrimidine	NCGC00123034		35.2 ± 4.52	32.1 ± 12.6	–	0.92
Cluster 8 1 2 3 4-tetrahydroacridine	NCGC00110901		10.6 ± 1.32	36.1 ± 15.2	–	3.41
Cluster 9 thieno[2,3-d]pyrimidine	NCGC00104044		38.7 ± 57.9	9.94 ± 0.93	–	0.26
Cluster 10 10,11-dihydrodibenzo[b,f][1,4]thiazepine	NCGC00140326		~60	13.8 ± 0.82	–	0.23

IC_50_, mean half-maximum inhibitory concentrations determined from experiments against *P. falciparum* NF54 gametocyte, asexual parasite and HepG2

– Indicates compounds with less than 25% cytotoxicity at up to 46 µM

^a^ In HepG2 cytotoxicity assays, NCGC00114940 showed 35.7% response at 15.3 µM and 33.3% response at 46 µM. Data are presented as mean ± SD with n = 3

### Activities of confirmed compounds against asexual parasites

In order to identify the compounds active against both gametocytes and asexual parasites, the activity of the 32 confirmed gametocytocidal compounds was determined using a parasite growth inhibition assay [27]. Among the compounds tested, 27 of them inhibited the growth of asexual parasites with IC_50_s less than 10 µM and maximum responses >75% (Table 1; Fig. 2). The remaining five compounds showed IC_50_s higher than 10 µM (Table 1). Three compounds (NCGC00134126, NCGC00134124, and NCGC00110901) showed slightly lower IC_50_s in the gametocyte viability assay than that in the asexual parasite growth assay (ratio of IC_50_s = ~fourfold) and 9 compounds (NCGC00134795, NCGC00100599, NCGC00101506, NCGC00141020, NCGC00100597, NCGC00127015, NCGC00126987, NCGC00104044, and NCGC00140326) showed higher IC_50_s in the gametocyte viability assay than that in the asexual parasite growth assay (ratio of IC_50_s from 3.21 to 17.5-fold). The other 20 compounds showed similar activity (ratio of IC_50_s within threefold). Among these confirmed hits, 16 compounds decreased gametocyte viability to <10% in the preparations of late stage gametocytes, including the 3 molecules with an IC_50_ gametocyte/asexual ratio of ~4.

### Cytotoxicity of confirmed compounds in HepG2 cells

To eliminate the general cytotoxic compounds from the confirmed compounds, the cytotoxic effect of these compounds was determined in mammalian HepG2 cells with an AlamarBlue assay using an 11-concentration titration from 0.001 to 46 µM. Of the 32 compounds tested 28, including the 3 compounds that preferentially targeted gametocytes, were not cytotoxic at the highest compound concentration of 46 µM in the HepG2 cells. The remaining four compounds showed weak cytotoxicity with respect to HepG2 cells: NCGC00127017 (IC_50_ = 25.32 ± 5.41 µM, maximum response at 87.6%), NCGC00127015 (38.39 ± 0.48 µM, maximum response at 87.2%), NCGC00126987 (38.6 ± 0.42 µM, maximum response at 86.1%), and NCGC00126892 (34.37 ± 5.36 µM, maximum response at 93.0%).

### Three novel structural clusters identified from confirmed active compounds

The chemical structures of the confirmed compounds were then analysed based on their chemical similarity and found 10 diverse clusters (Table 1). Several structural clusters are known anti-malarial scaffolds including 2,4-diaminopyrimidines (for example, NCGC00134128, pyrimethamine-like), 4H-chromen-4-ones (for example, NCGC00100599, MMV665820-like) [4], and 8-quinolinols (for example, MMV000788-like). 4H-chromen-4-ones are more active against sexual parasites than against asexual parasites. Notably, three novel clusters containing 3-amino-imidazo[1,2-a]pyridines (for example, NCGC00104490), 3H-imidazo[4,5-b]pyridines (NCGC00134795), and 4-1H-pyrazol-5-yl piperidines (for example, NCGC00127017) were active against both asexual and sexual parasites. To the best of our knowledge, these new scaffolds have not been reported as anti-malarial agents before.

### Human kinase profiling of the two most potent hits

**Fig. 3:**
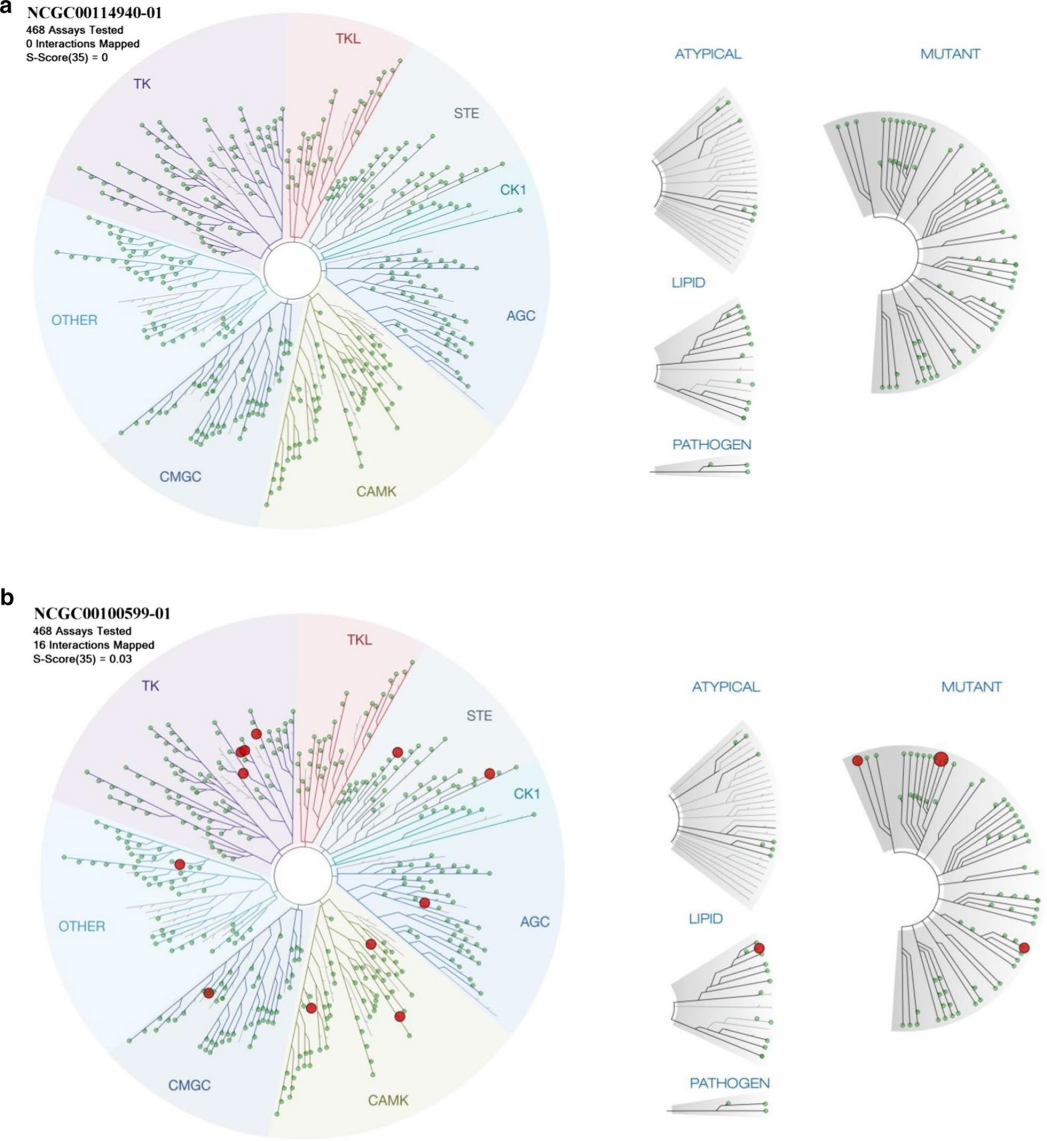
Human kinase profiling of NCGC00114940 and NCGC00100599. 10 µM NCGC00114940 (**a**) and 10 µM NCGC00100599 (**b**) were tested against 468 human kinases for binding activities, respectively. NCGC00114940 did not interact with any tested human kinases. NCGC00100599 may interact with 16 human kinases. The *top three* targets are ABL1 (Q252H)-phosphorylated (9.9% of control), PIK3CD (10% of control), and CDK4-cyclin D3 (15% of control)

To avoid compounds that also inhibit human kinases, as has been a problem for some previous anti-malarial drug candidates [14, 28], the binding affinities to 468 human kinases were tested for the two most potent compounds identified in this screen. NCGC00100599 is the most potent compound against asexual parasites with an IC_50_ of 0.339 µM and a gametocytocidal IC_50_ of 4.18 µM. The other compound NCGC00114940 was similarly effective against gametocytes (IC_50_ = 0.620 µM) and asexual parasites (IC_50_ of 1.16 µM). At 10 µM, NCGC00100599 had relatively low affinity inhibitory activity against 13 out of 468 human kinases (Fig. 3; Table 2), whereas NCGC00114940 did not affect the activity of any of the 468 human kinases (Fig. 3).

**Table 2:** Kinome scan profile of NCGC00100599

Kinase	% Control	Kinase	% Control
ABL1(Q252H)-phosphorylated	9.9	RSK2(Kin.Dom.2-C-terminal)	29
PIK3CD	10	IKK-epsilon	31
CDK4-cyclinD3	15	JAK2(JH1domain-catalytic)	31
ERBB3	17	MYLK	31
TYK2(JH1domain-catalytic)	19	PRKG2	32
TRKA	22	KIT(A829P)	34
SNRK	27	MAP3K3	34
ALK(C1156Y)	28	MST4	34

NCGC00100599 was profiled at 10 μM against a diverse panel of 468 kinases by DiscoverX

## Discussion

Ideally, the next generation of anti-malarial drug combinations will treat clinical symptoms and eliminate malaria transmission [29]. This target profile will require new small molecules with efficacy against gametocytes with dual activity against asexual blood stages. In this work 45,056 diverse compounds were screened for gametocytocidal activity. In the hit confirmation experiments, the activities of primary screening hits were determined in both gametocyte assay and asexual parasite assay. Three compounds were identified that were slightly more active against gametocytes than asexual parasites and an additional twenty compounds had dual-efficacy against both gametocytes and asexual parasites with similar level of potencies. The consistent strong gametocytocidal activity against preparations of late stage gametocytes containing 30–60% stage V gametocytes in the experiments strongly suggests the efficacy against the mature stages directly responsible for transmission. To precisely define the relative activity against male and female gametocytes, additional analysis is needed [15].

The structural analysis of the 23 confirmed compounds found they could be divided into 10 scaffold clusters, three of which were not previously associated with anti-malarial compounds or to any known anti-malarial scaffolds. Within these three new scaffolds, 3-amino-imidazo[1,2-a]pyridines were reported as PGHS-2 inhibitors with analgesic and anti-inflammatory activities [30]. 3H-imidazo[4,5-b]pyridines were inhibitors of luciferases used as reporter enzymes [31]. Interestingly, 4-1H-pyrazol-5-yl piperidines have been classified as ‘dark chemical matter’(DCM) recently [32]. The term DCM is used to refer to a collection of small molecules which have not shown any biological activity although these molecules have been extensively tested in a variety of high throughput assays. Hit molecules coming from DCM may provide high selectivity and clean safety profiles with minimum off-target toxicity [33]. Due to the intrinsic DCM characteristics of 4-1H-pyrazol-5-yl piperidine analogs, they could serve as a valuable starting point for the medicinal chemistry campaign for drug development.

Drug safety is critical for development of the next generation malaria agents [4]. The ideal compounds for development should have selectivity against targets in *Plasmodium falciparum* parasites without significant cytotoxicity to mammalian cells. Inhibition of a few of human kinases, such as PI4KB [3], mTOR [14], and PI3K [34] by several previous anti-malarial compounds has been reported [14] and will need to be avoided for the new generation of anti-malarial drugs. In the lead optimization studies, these activities against human kinases need to be removed, while the anti-malarial activity remains. The most potent gametocytocidal compound NCGC00100599 found in this study did not bind to any of 468 human kinases tested at 10 µM compound concentration. These 468 human kinases include AGC, CAMK, CMGC, CK1, STE, TK, TKL, lipid and atypical kinase families, plus important mutant forms [35, 36]. The kinase profiling results suggest that the lead compound has no relative impact on human kinase targets.

## Conclusions

In summary, a set of novel compounds with dual-efficacy against both gametocytes and asexual parasites were identified from a high throughput screening of 45,056 compounds in the gametocyte viability assay. The structural cluster analysis identified three novel classes of structures in the confirmed compounds. The 4-1H-pyrazol-5-yl piperidine analogs were highly selective against malaria parasites without activities in the 468 human kinases tested. These confirmed compounds provide potential starting points for future anti-malarial drug development.

## Additional file

**Additional file 1.** A Giemsa-stained smear of one of the late stage gametocyte preparations.
