# Supplementary figures and images for: Novel lead structures with both Plasmodium falciparum gametocytocidal and asexual blood stage activity identified from high throughput compound screening
Source: Malar J. 2017 Apr 13;16:147. doi: 10.1186/s12936-017-1805-0 (PMC5390467; doi:10.1186/s12936-017-1805-0)

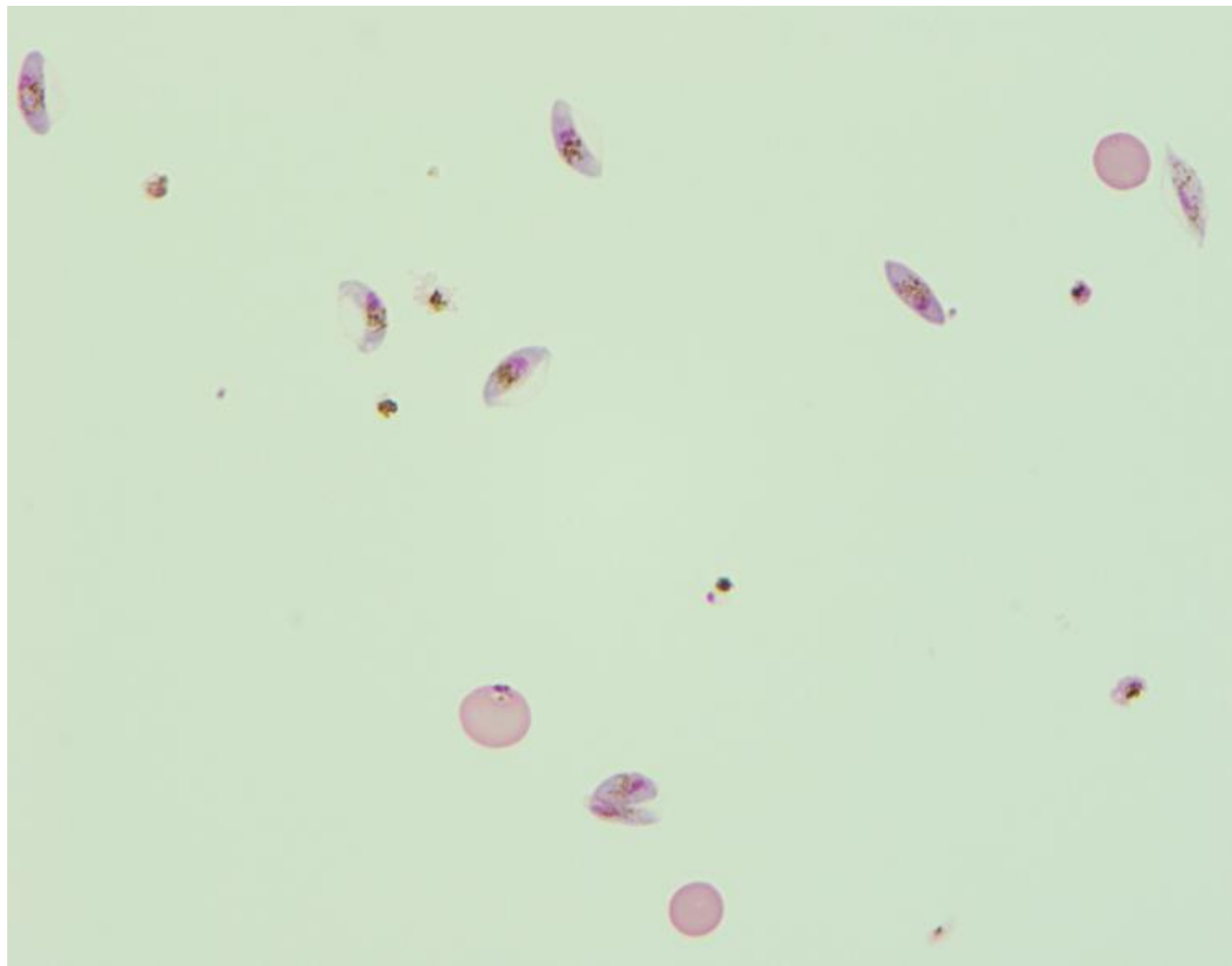

Supplement: Supplementary file 1 — Additional file 1. A Giemsa-stained smear of one of the late stage gametocyte preparations. [file 12936_2017_1805_MOESM1_ESM.pdf]
